# Health impact of borders: general reflections and a case study from the Polish–German border

**DOI:** 10.1007/s10198-023-01588-5

**Published:** 2023-04-12

**Authors:** Julia Kuntosch, Marie-Luise Ruebsam, Jakub Orsson, Dorota Orsson, Klaus Hahnenkamp, Jörg Hartleib, Steffen Flessa

**Affiliations:** 1 Department of Healthcare Management, University of Greifswald, Friedrich-Loeffler-Strasse 70, 17487 Greifswald, Germany; 2 Department of Anesthesia, Intensive, Emergency and Pain Medicine, University Medicine of Greifswald, Ferdinand-Sauerbruch-Strasse, 17475 Greifswald, Germany; 3 Department of Geography, University of Greifswald, Friedrich-Ludwig-Jahn-Strasse 16, 17487 Greifswald, Germany

**Keywords:** Border, Catchment area, Cross-border healthcare, Distance decay effect, Polish–German border area

## Abstract

**Background and objective:**

Political, economic, communicative and cultural borders still limit the accessibility of acute healthcare services for patients so that they frequently have to accept longer distances to travel to the next provider within their own country. In this paper, we analyze the impact of borders and opening of borders on acute medical care in hospitals and on patients in border regions.

**Methods:**

We develop a conceptual framework model of cross-border healthcare and apply it to the Polish–German border area. The model combines the distance decay effect, a catchment area analysis, economies of scale and the learning curve.

**Results:**

Borders have a major impact on acute medical care in hospitals and on patients. Setting of new borders will reduce the accessibility of health facilities for patients or require the establishment of new hospitals. Reopening borders might induce a vicious circle leading to the insolvency of a hospital which might result in poorer health for some patients.

**Conclusion:**

Strong effort should be invested to overcome political and cultural borders to improve the health of the population in border regions. Similarly, increased cross-border acute healthcare must be seen in the context of rural health and the special situation of small rural hospitals in rural peripheral areas.

## Introduction

The Schengen Agreement (14 June 1985) abolished internal border checks within the Schengen Area consisting of 26 European countries (year 2022). Consequently, cross-border cooperation strongly increased, and cross-border friendship, trade, tourism, and shared cultural activities have become (almost) as normal as within a single country [1–3]. While most of these activities are based on private funds and engagement, cross-border acute healthcare usually requires quite some Governmental collaboration and regulations as well as financing agreements [4]. Still, cross-border healthcare has also become a routine between several countries. For instance, patients from Luxembourg are admitted to German and French hospitals, while ambulances from Luxembourg serve patients from their neighbouring countries [5].

The starting point was the “EU-Directive 2011/24/EU of the European Parliament and the Council of 9 March 2011 on the application of patients’ rights in cross-border healthcare”. It regulates that every citizen of the EU has the right to use the health services in any EU country. For instance, a Polish woman has the right to deliver her child in a German hospital and a German patient living close to the border must be accepted by a Polish hospital. The patient will be refunded for the expenditure in the other country by his own financing mechanism (usually health insurance), but the amount will be not higher than the respective costs within the country of residence. For instance, if a Polish woman delivers her child in a German hospital, she will have to pay the G-DRG-rebate (O60D) but will be refunded less than 20% (year 2023) of this amount by her Polish health insurance and has to bear the difference herself. Only in the case of an emergency, i.e., when she has no chance to travel back to her own country, the full costs will be refunded [6].

If two neighbouring countries have almost the same gross national product per capita (e.g., Euro-Region Rhine-Waal), the costs covered by the respective health insurance funds will be roughly the same so that the EU Directive 2011/24/EU guarantees that EU citizens have a choice of the provider of healthcare services on both sides of the border. They can, for instance, travel to the closest provider even if it is in the other country. The implementation merely requires some administrative effort for transfer of refunds. However, if the economic strength and in particular the income per capita are quite different between neighbouring countries (e.g., between Poland and Germany), the choice of the healthcare provider is limited. The ability and willingness to pay for the non-refunded part of the cost in the richer country prevents patients from seeking healthcare in the location with the highest utility for them.

The analysis of catchment areas is a standard in economic geography since Christaller published his fundamental work on centrality in Southern Germany in 1933 [7]. Later, the principles were applied to cross-border trade und border regions, e.g., [8–10]. However, most of this work does not focus on health and in particular on the impact of borders on healthcare. There is a growing literature on legal conditions of cross-border healthcare [11–13] and some case-studies of particular borders without explanation of the negative role of borders [14, 15]. Recently, cross-border emergency care attained more attention during the COVID-19 pandemic. Some countries closed their borders; others transported patients from hot spots across the border to countries with lower incidence [16, 17]. However, this work does not explain the role of borders in acute and emergency medical care beyond the example. Thus, the impact of borders on acute medical healthcare and health has not been sufficiently analysed.

Most studies with a focus on cross-border healthcare stress the relevance of administrative and communication cultural barriers but do not reflect on the general effect of political, financial or cultural barriers on the health of citizens from an economic perspective [18]. In this paper, we would like to close this research gap by developing a model of cross-border healthcare with a focus on acute and emergency care and applying it to the Polish–German border. In the next section, we develop the conceptual framework model based on the distance decay effect and a catchment area analysis as well as on economies of scale and the learning curve. Based on this model, we demonstrate the impact of borders on healthcare and health. In the fourth section, we describe the situation in the border area between Poland and Germany to demonstrate the relevance of the framework model. The paper closes with some conclusions for policymakers.

## Conceptual framework

In this section, we develop a conceptual framework model that allows analysing the impact of borders on the demand for healthcare services. The model is based on four basic concepts: the distance decay effect, a catchment area analysis, the economies of scale and the learning curve.

### Distance decay effect

**Fig. 1:**
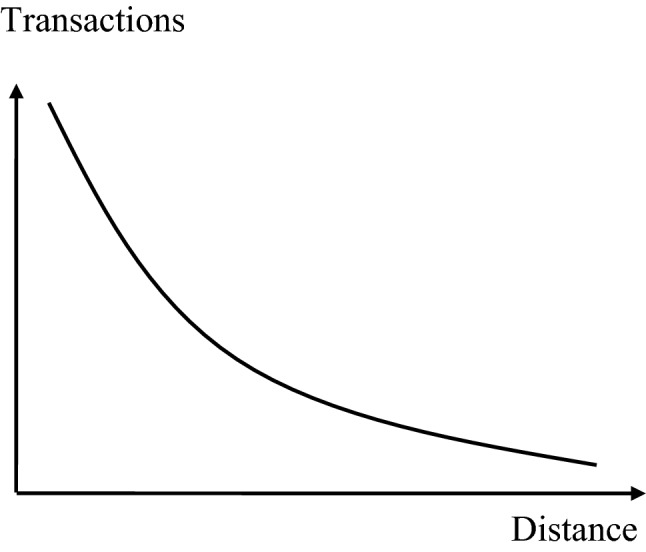
Distance decay effect. Source: [19]

The distance decay effect describes the relationship between the distance between two centres and the number of transactions between them [19]. As Fig. 1 exhibits, the number of transactions declines with increasing distance. For instance, most patients visit a medical expert more often in their own town than in a neighbouring town, and they use the services of a medical doctor in the neighbouring town more frequent than in the capital city. The gravity between two places depends on the attraction of each of these places, the distance between these places and the friction of distance, i.e.,

$$G\approx \frac{{A}_{1}\cdot {A}_{2}}{{d}^{f}}$$

With *G* gravity between two places, *A*_*i*_ attraction of place *i*, *i*=1,2, *d* distance between place 1 and place 2, *f* friction of distance constant

The attraction of a central place expresses how strongly the place pulls people to get into interaction with it. For instance, a bigger town with a broader portfolio of services, shops and facilities attracts more people than a smaller town without these facilities. A bigger hospital with high technology medicine and sub-specialisation will attract more patients than a small hospital of primary care. In principle, the attraction of a hospital is not proportional to the number of beds but to the level of services provided. However, in reality, size and function of a hospital are highly correlated, i.e., tertiary hospitals with high quality services do usually also have more beds than primary hospitals [20].

The distance between two places (usually expressed in km) can be perceived quite differently and will have a different impact on the decisions of people. For instance, a functional infrastructure of public transport reduces the impact of distance on gravity between two places, and mental mobility decreases the impact even further. Therefore, distance *d* is weighted with the friction constant *f*. If *f* is high, the distance will have a higher impact on the gravity between the two centres. Furthermore, the distance decay depends on the service offered in a particular location. For instance, a distance of 10 km might be too long to seek preventive dental care, while even 50 km might not be insurmountable for acute dental care with tooth pain, i.e., the distance decay for acute and in particular emergency care is lower than for planned care.

### Catchment area analysis

**Fig. 2:**
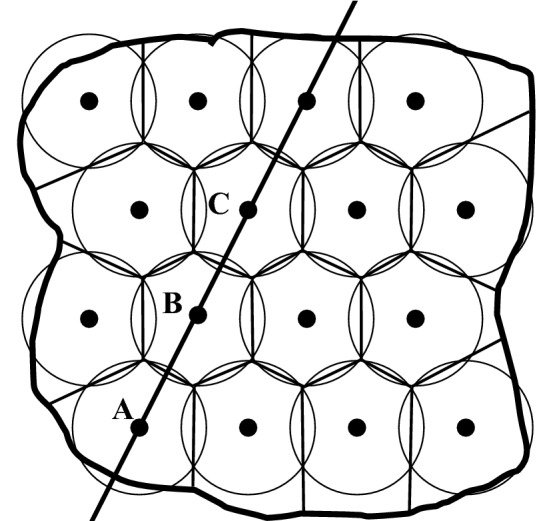
Development of hexagons. Source: [21]

The catchment area analysis assumes a homogenous space where the population is equally distributed. The objective is to minimize the average distance between each citizen and a central service provider. As demonstrated [21], hexagons are the ideal geographical pattern to cover the entire space and population with service centres (see Fig. 2).

**Fig. 3:**
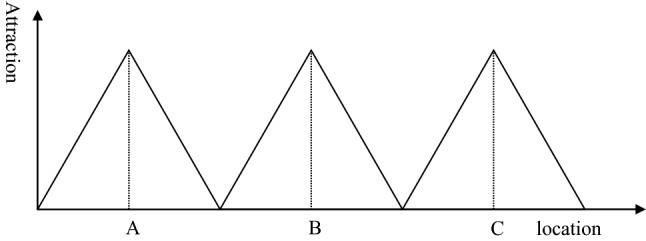
Basic model. Source: [21]

To analyze the impact of borders on the catchment area and service availability, it is sufficient to reduce the two-dimensional analysis (area) of Fig. 2 to a one-dimension analysis (line) of Fig. 3. Thus, the *x*-axis of Fig. 3 represents Fig. 2 along the bold line, while the *y*-axis represents the attraction of each centre. For the basic model, we assume that three healthcare providers (A, B, C) are on the bold line. They provide the same level of care and have roughly the same size, so that their attraction is rather similar. We assume a linear distance decay effect, i.e., the attraction decreases with a constant rate. In the basic model, there is no place where none of these hospitals is attractive to patients, but the attraction lines do not intercept. Furthermore, we assume that these hospitals can break-even, i.e., the number and severity of patients are sufficient to recover the costs under the given financing mechanism. Thus, the basic model represents an ideal situation: the entire population is covered by hospital services and each hospital can be sustained.

### Economies of scale

The economies of scale are a basic economic principle stating that a higher output decreases the cost per output unit (unit cost). Furthermore, big organisations usually have lower unit cost than smaller organisations. The underlying assumption is that most organisations have fixed cost (cost that do not increase if the output increases, such as cost of buildings, equipment, basic staff…). If the output increases, the fixed cost per output unit decreases. Consequently, the cost per output unit will decrease with increasing output.

**Fig. 4:**
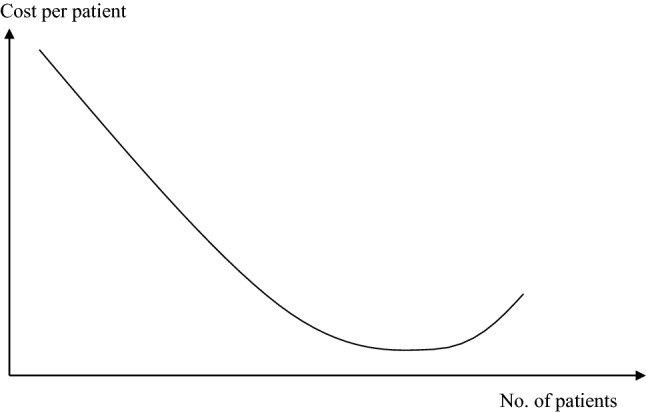
Unit cost of a hospital. Source: [22]

This principle can be applied to healthcare organisations, such as hospitals. Figure 4 shows that the unit cost declines with increasing number of patients. Whether a hospital has 100, 150 or 200 patients, it will still need one radiographic system, one laboratory and one administration. Thus, the overheads of the hospital are divided between more patients in bigger hospitals. However, very big hospitals have a tendency of developing complex and intransparent organisational system resulting in increasing costs at the right tail [22, 23].

The figure does not consider step-fixed costs, and it only indicates (right tail) that there can also be diseconomies of scale. But in principle it is generally accepted that bigger hospitals have lower costs per case than smaller hospitals if other variables (e.g., quality of services) are constant [24].

### Learning effect

**Fig. 5:**
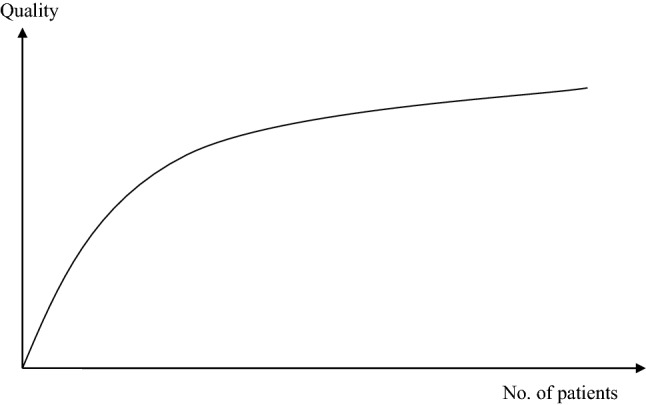
Quality and volume. Source: [28]

The quality of a healthcare service strongly depends on the frequency with which it is performed [20]. This is true for the correct response to risks. For instance, a midwife might be able to manage 90% of deliveries without any problem even if she delivers only one child per week in a small hospital. But the rare event of a life-threatening complication will require that she has some routine in taking the right action [25–27]. Consequently, a number of studies have demonstrated a strong correlation between volume and outcome of medical procedures (Fig. 5) [28].

## Health impact

Based on this model, we demonstrate the impact of borders on acute medical care and on the health of citizens in neighbouring countries. We assume that negative consequences can have different dimensions:

- *Poor coverage*: If the hospitals are too far from each other so that the attraction lines do intercept below y = 0, a part of the population is not attracted by any hospital, i.e., the distance between their place of living and the next service provider is too high. This is a consequence of the distance decay effect (see Sect. “Distance decay effect”). If we assume a linear distance decay curve as shown in Fig. 3, the maximum travel distance to a health facility is the distance where the gravity of the hospital is zero.
- *Quality of services*: The quality of healthcare services frequently depends on the frequency of procedures (learning effect, see Sect. “Learning effect”). Thus, reduced demand for healthcare services will result in poor quality of services. A higher quality in a location will lead to a higher attraction. However, quality is mainly subjective quality because the perception of the quality of services determines the attraction to the customer/patient, not the objective quality [29]. Thus, ability to communicate in the language of the customer, understand his culture and give access to the network (e.g., relatives visiting the patient in hospital) are elements of the quality of a central location and consequently determine its gravity.
- *Poor health*: Poor coverage will result in poor demand for healthcare services, little service and finally in poor health of the population in the under-served areas. This is an assumption that is not based on the conceptional framework of Sect. “Conceptual framework”, but a general assumption that the health of the population depends on the availability and accessibility of healthcare services.
- *Financial loss*: If the demand for healthcare services declines, the cost per service unit will increase (economies of scale, see Sect. “Economies of scale”) so that the risk of not recovering the costs increases. This might lead even to bankruptcy of the hospital.

**Fig. 6:**
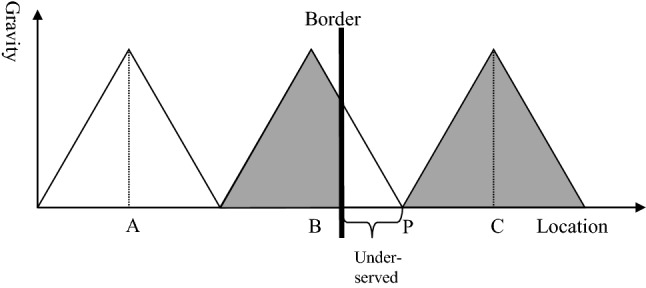
Service gap resulting from a border. Source: Own

Figure 6 shows the impact of a border between hospital B and C. If the border was just in the middle between both hospitals (point P), it would not have an impact on the catchment areas and on the health of the population. However, in reality borders do not follow the line of nearest distance between two service centres. The black line represents the border and is closer to hospital B than to hospital C. Consequently, the catchment area of hospital C remains unchanged while the catchment area of hospital B is reduced. This has two consequences. Firstly, the population living between the border and point P is underserved. They would feel attracted by hospital B, but the border prevents them from going there. At the same time the population in this underserved area lives too far away from hospital C, i.e., it is not attracted by this hospital. Thus, the first consequence of establishing a border is an underserved area with severe consequences for the health of the citizens residing in this area.

The second consequence is financial hardship for hospital B. The hospital has a certain capacity based on its original catchment area. After introducing the border, the catchment area declines so that the hospital might enter a vicious circle. A smaller catchment area with less patients means that the hospital will have higher costs per patient (economies of scale) and lower quality (learning curve). The management of the hospital will answer to this development by reducing agents of production (e.g., staffing, leading to lower level of quality) and/or scope of services. This will result in a lower attraction for patients, and this will again reduce the catchment area.

**Fig. 7:**
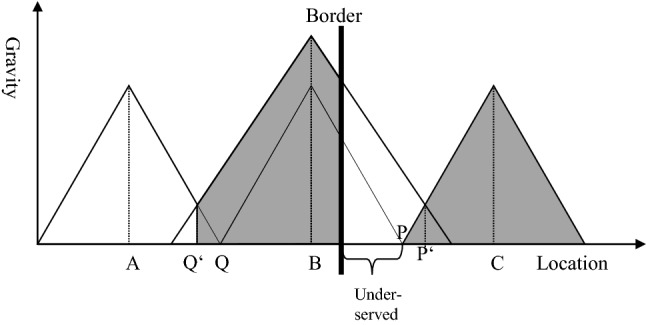
Service gap resulting from a border, hospital B with higher quality. Source: Own

Figure 7 shows the situation if hospital B has a higher quality than the other hospitals. As stated before, a higher quality means a higher attraction at any place. Consequently, the catchment area of hospital B increases from Q to Q′. Without a border, the higher quality of hospital B would result in an increase of the catchment area from P to P′. However, because of the border, the real catchment area of hospital B goes only from Q′ to the border. On the other side of the border, the region between the border and P is underserved, just as shown in Fig. 6. The population between P and P′ is still attracted by hospital C, but less than by hospital B. Thus, a situation where the hospital on the other side of the border has a higher quality (and consequently attraction) will not increase the underserved area. Nevertheless, the share of the population that is in a worse healthcare situation than without the border increases if the neighbouring hospital has a higher quality.

**Fig. 8:**
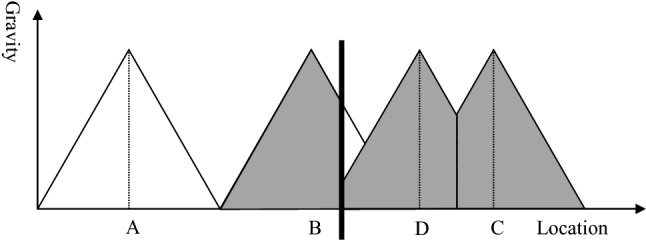
Establishment of a new hospital. Source: Own

Figure 8 exhibits the situation of the catchment area after building a new hospital. Hospital D covers the gap between hospital B and C to compensate for the problems arising from the establishment of the border between the two countries. Thus, there is no more under-served area, expressed in the graph by the fact that the attraction of a hospital is greater than zero for any location. However, the catchment area of hospital D and C is smaller than in the original model without a political border (see Fig. 3). Consequently, the hospitals will face economic challenges. With the reduced catchment area, their unit costs will be higher and the quality most likely lower than in the standard without borders. Thus, establishing a new hospital might make hospital services available in any place, but it induces economic stress and might result in poor quality of healthcare services for the population. Thus, the establishment of borders is likely to result in poor health for the population irrespective of whether border areas remain underserved or whether new hospitals are established. As poor healthcare regularly induces poor health, one could say that borders kill.

Figures 6 and 8 exhibit that political borders have a negative impact on the health of the population because people cannot choose the nearest hospital, new hospitals must be established, and the remaining catchment areas are too small to sustain the facilities. This can induce a vicious circle of poor occupancy, high unit cost, poor quality and again poor demand for acute healthcare services. This statement alone should be sufficient to convince decision-makers to open borders and make a joint cross-border healthcare planning in particular for acute and emergency medical care. However, central healthcare planning of Governments is very territorial, i.e., borders are seen as impenetrable walls and the area behind the wall is considered as completely irrelevant for the central planning of the own country. There is an urgent need to convince politicians and ministerial officers that borders reduce the quality of life of their citizens.

Officially, the borders in Europe since the Schengen treaty are open, and the border between Poland and Germany became highly permeable on 1st of May 2004 when Poland jointed the Schengen area. However, until today hardly any patient from these two countries seeks acute healthcare in the respective other country [30]. People are usually willing to travel long distances to a hospital on their own side of the border.

Several reasons have been discussed. The main barriers are the different languages, (perceived) quality standards, culture, and cost. In the following, we will discuss the long-term impact of opening border for the hospital infrastructure on both sides. Therefore, we have distinguished three different scenarios.

**Fig. 9:**
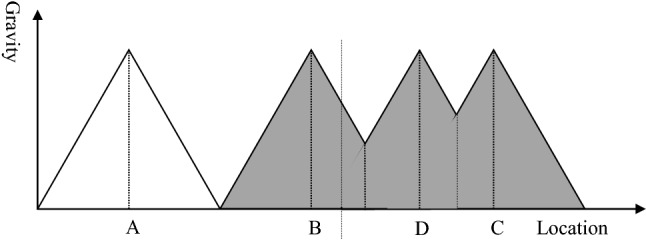
Opening borders, same income level. Source: Own

The first scenario are countries of the same level of income (e.g., between France, Luxembourg, and Germany). Figure 9 shows the situation after opening the border when both countries have (roughly) the same level of income. In the beginning, hospital D is sustained, but the catchment areas of hospital D are reduced while the catchment area of hospital B increases. The model assumes that patients go to the nearest hospital, i.e., where the gravity is highest. Based on the distance decay effect (see Sect. “Distance decay effect”) patients closer to hospital B beyond the border will prefer hospital B after opening the border if no other barriers persist.

Thus, the new situation is more favorable for the patients because distances for the patients are lower. However, the occupancy rate of hospital D declines as well leading to increased unit costs (economies of scale) and decreased quality of care (learning effect). Hospital D will get under stress and might have to be closed so that we will have the original situation as it was before the border was established (see Fig. 3). After closing hospital D, hospitals B and C take over the respective catchment area and serve the population.

**Fig. 10:**
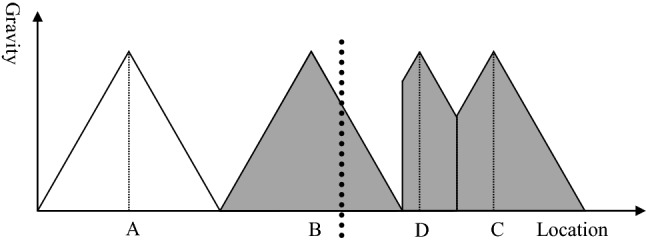
Opening borders, different income level and full cost recovery. Source: Own

**Fig. 11:**
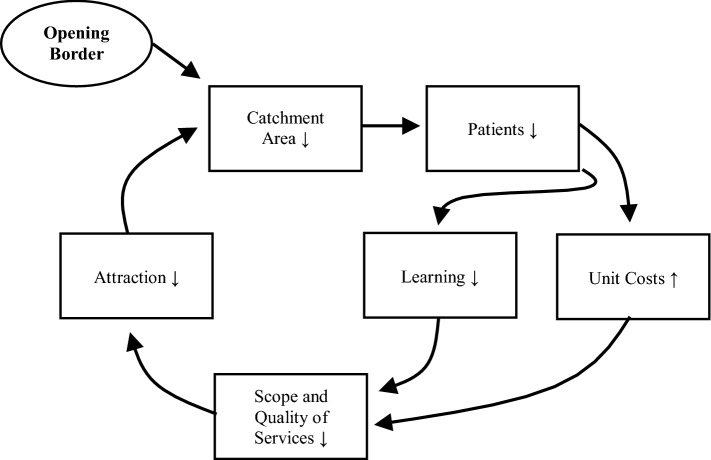
Vicious circle initiated by opening a border. Source: Own

Scenario 2 assumes that the countries have different income levels, but the costs of treatment in the other country are refunded fully. Figure 10 assumes that the country on the left of the border is richer than the country on the right. In this case, hospital B is in the richer country and will have a higher quality than hospital C and D in the poorer country. Thus, some people living closer to hospital D than B will still attempt to use the services of hospital B with the higher quality. This can induce a vicious circle in hospital D (see Fig. 11) that finally might lead to insolvency and closing-down of a hospital: a reduced catchment area means lower number of patients, which induces higher unit costs and poorer quality. The management can only answer to this challenge by reducing the scope and quality of services, which will decrease the attraction of the hospital again. The circle presented in Fig. 11 might result in the insolvency of hospital D. However, the necessity to close the hospital is not a consequence of poor management but of an autocatalytic process initiated by opening a border. Eventually, we will come back to the original situation before establishing a border (see Fig. 3).

**Fig. 12:**
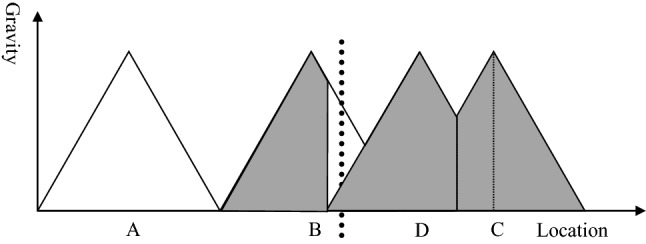
Opening borders, different income level and no cost recovery. Source: Own

The third scenario represents the situation where the border between two countries with different income is open, but costs are not recovered, i.e., the patient from the poorer country (here: right of the border) will have to pay the cost of acute healthcare in the other country out-of-pocket. Thus, the number of patients seeking acute healthcare from the country with the lower income in the country with the higher income is exceedingly small. Instead, patients from the country with the higher income will seek services in the poorer country (hospital D). Services which are not included into the basic healthcare package of the richer country so that they will have to be paid out-of-pocket (e.g., dentures, in-vitro-fertilization) will be demanded by citizens from the richer country in the poorer country. At the same time, health insurances from the richer country might support their members to seek healthcare services in the country with the lower costs. In both cases, opening the border might weaken hospital B in the richer country as shown in Fig. 12.

In this case, we have to distinguish between different social groups and different health goods resulting in different gravity lines. For instance, the richer part of the population of the poor country might seek acute healthcare services in the richer country and pay out of their own pocket. Thus, their distance decay differs strongly from the respective effect of the poorer population unable or unwilling to pay for healthcare services in the neighboring country out-of-pocket. Thus, the slope of the (here: linear) gravity function has to be distinguished between groups. The population on the richer side of the border would, most likely, only seek specialized healthcare services on the other side of the border, in particular services which are not covered by their own health insurance in their own country and which are cheaper in the poorer country, such as in-vitro-fertilization or dentures. Again, the gravity functions will differ between goods.

The scenario is relevant for the analysis of medical tourism [31, 32]. This term was originally used for rich minorities travelling to major centers in richer countries with a high quality of services, such as Arabic citizens travelling to US-hospitals for treatment. However, the term is also relevant for patients crossing the border between neighboring countries if the services are better on the other side. Furthermore, the term is meanwhile used for patients who travel to countries for cheaper medical treatments. Most of them are, however, planned treatments. Acute and in particular emergency care is usually not considered as an element of medical tourism. It is, nonetheless, highly relevant in the border area between two countries.

Finally, a fourth scenario covers the case that the hospital on the richer side of the border has a higher quality than the hospital on the poorer side. Based on the other scenarios, we can assume that this hospital will—ceteris paribus—attract even more patients from the other side of the border if they can afford it. However, we have to consider that the attraction of a hospital is mainly based on subjective quality, which included aspects like communicating in the language of the patient, understanding his culture, giving access to the relatives to visit him and generally feeling welcome. Thus, it could be that the hospital on one side of the border has a higher objective quality while the subjective quality is lower than on the own side of the border because of these inter-cultural limitations.

## Case study: Polish–German border

**Fig. 13:**
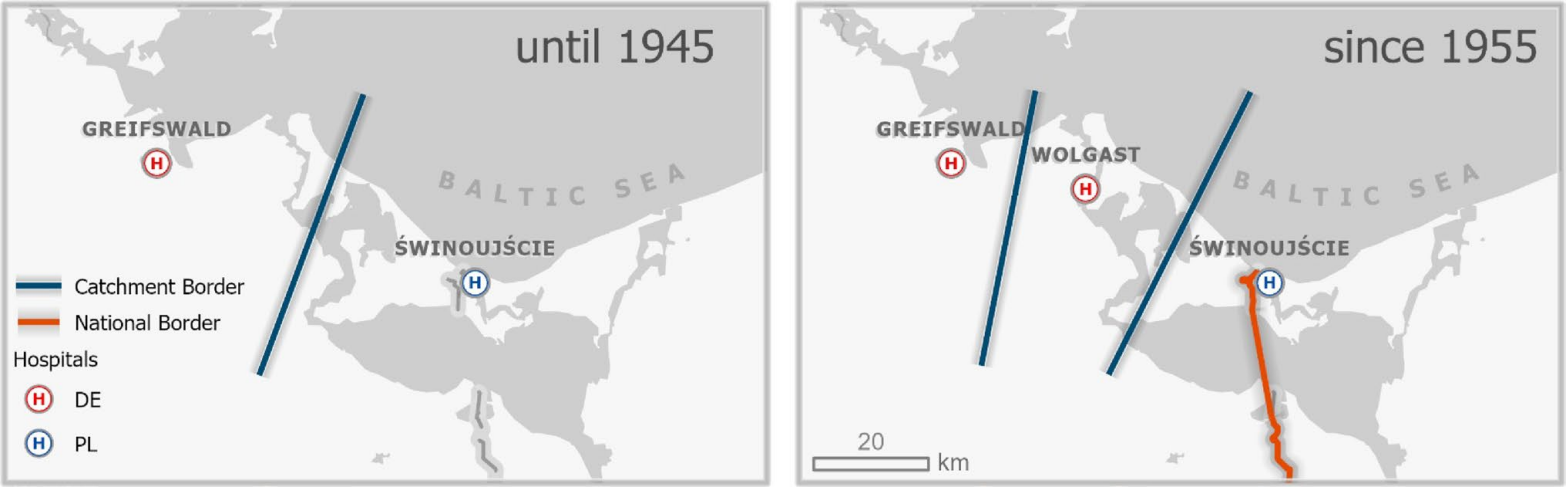
Shortest distance catchment areas on Usedom Island. Source: own

As shown in Sect. “Health impact”, setting of borders challenges the existing hospitals and requires the establishment of new facilities. This happened in several locations at the (then) new border between Poland and Germany after World War II. For instance, the island of Usedom was serviced by the hospital in Swinoujscie in the East of the island. Toward the west, the next hospital was in Greifswald, but as Fig. 13 shows, the biggest part of Usedom Island is closer to Swinoujscie than to Greifswald. The bold line on the left of Fig. 13 represents the boundary of the shortest distance catchment area between Greifswald and Swinoujscie Hospital reflecting a rather ideal situation as in Fig. 3. After the political border (red line close to Swinoujscie) separated the biggest part of the population of Usedom from their traditional hospital in Swinoujscie, the healthcare situation became difficult and close to the situation of Fig. 6. The distance between Greifswald and the last village to the border to Poland is about 65 km, i.e., a very long distance in a situation of poor public transport. Everybody between the bold line on the left of Fig. 13 and the political border between Poland and Usedom had to travel higher distances to visit a hospital—sometimes too high to accomplish.

Consequently, the Government of the German Democratic Republic decided to build a new hospital in Wolgast in 1945 that still exists until today. It reflects precisely the situation exhibited in Fig. 8. Consequently, the hospital in Swinoujscie lost a major part of its catchment area and was too big for the remaining population, while the hospital in Greifswald was too far away for the population in Usedom so that a new hospital had to be built to avoid an under-served area. After opening the borders again, acute care remained quite territorial as the financial and cultural burden still constitute a border as presented in Fig. 12.

**Fig. 14:**
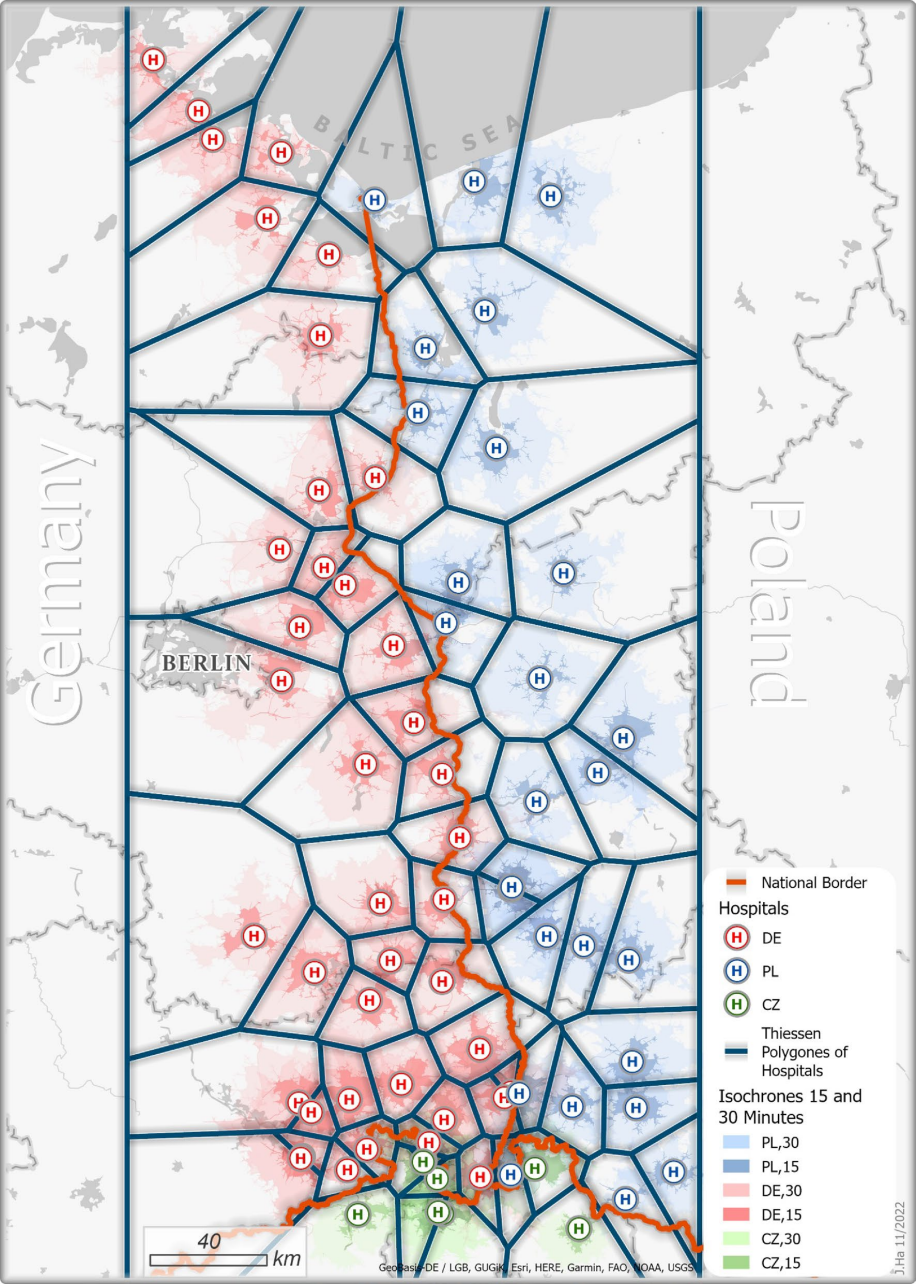
Thiessen-Polygons of hospital catchment areas in Poland and Germany. Source: own

As Fig. 14 demonstrates, the situation of the hospital catchment areas on the island of Usedom is no unique exemption. The Figure shows the so-called a Voronoi diagram with several Voronoi cells (Thiessen-Polygons) consisting of all points of the plane closer to the centre of that cell than to any other, i.e., the polygon represents the shortest-distance catchment area [33]. In many places, the border divides the shortest distance catchment areas so that people have to travel for longer distances to get to the next hospital on their side of the border. After Poland joined the Schengen Area in 2004, one would have expected that cross-border acute healthcare would have increased, in particular for emergency patients where access time is crucial and selecting the nearest hospital as destination of the emergency operation can mean literally a question of life-or-death. However, an analysis of cross-border emergency operations in the region of Vorpommern-Greifswald in the years 2015–2020 indicates (see Table 1) that cross-border emergency services are still a rare event.

**Table 1:** Cross-border emergency operations in the region of Vorpommern-Greifswald from 2015 to 2020. Source: [35]

Emergency site	Destination	2015	2016	2017	2018	2019	2020	Total
Germany	Hospital in Poland	18	14	13	16	16	7	84
	Border	2	1		2		1	6
Poland	Hospital in Germany			1				1
Border	Hospital in Germany	20	7	2	5	2	4	40
	Hospital in Poland				1	1		2
Transfer from German hospital	Hospital in Poland		1		1	1	1	4
	Private address in Poland						2	2
Transfer from Polish hospital	Hospital in Germany	1		4	9	9	2	25
	Private address in Germany							

The table shows the number of emergency transports provided by the emergency services of Vorpommern-Greifswald differentiated by emergency sites and destinations as far as cross-border transfer is concerned. From the year 2015 to 2020 (latest data available), 84 patients were transported from an emergency site in Germany to a hospital in Poland. For six patients, the emergency site was in Germany and they were brought to the border where they were handed-over to a Polish emergency service. Only in one case, the emergency site was in Poland and an emergency car from Vorpommern-Greifswald came to provide services on the Polish side of the border.

For 42 patients the emergency site is recorded as “border”, but the documentation does not allow discriminating whether this was a hand-over of a patient at the border or whether the emergency was indeed at the border. 40 of them were brought to a German hospital, 2 to a Polish hospital. In addition, the emergency services of Vorpommern-Greifswald also handled a number of transfers of patients, i.e., they were transported from one hospital in Poland to a hospital address in Germany (25) or from a German hospital to a hospital/private address in Poland (6 patients). In comparison to the high number of emergency operations, cross-border emergency services are still a rare exemption, even 16 years after Poland joined the Schengen agreement.

We should add, that nationality and communication are a challenge even if the emergency site and transport destination are within one country as the patient might not be able to speak the respective language [34]. For instance, if a Polish citizens has an accident in Ahlbeck (just a lower distance from his home-country) and is transported by a German emergency service to the hospital in Wolgast, this is no cross-border emergency service, but still it might be a challenge for the emergency teams due to limited knowledge of the respective languages on both sides. Thus, borders may hazard effective emergency services even if they are not cross-border. In total, there were 198 emergency operation and 14 transfers with this language challenge from 2017 to 2020 (no data for 2015/2016).

## Conclusion

Based on the analysis of the situation of cross-border acute medical and in particular emergency healthcare between Germany and Poland we can conclude that borders still prevent people from seeking acute healthcare at the nearest provider. In the case of acute care and in particular of emergencies this can result in increased morbidity and mortality, i.e., borders are still likely to kill people. Under the Schengen Agreement, political borders do not really inhibit cross-border healthcare, but financial, political and communicative borders persist mainly in the mind of people. Patients fear to receive lower quality of care, meet staff who do not speak their own language or be treated inappropriately on the other side of the border (resulting in lower gravity as described in Sect. “Health impact”). The consequences of these mental borders are suboptimal locations of healthcare facilities, high investments in hospitals, longer access distances and times as well as medical risks.

This negative impact of borders is not reflected in the literature on medical tourism as it focuses on planned treatment in places of higher quality or lower cost. The need to reach the nearest place in case of emergency irrespective of the political territory is hardly reflected in health economic analyses and will require more research in future. Our models and the case-study from the Polish–German border region indicate that there is a major threat to the health of the population of border regions, but no comprehensive data base exists to assess the relevance and magnitude of this challenge. Consequently, we call for routine documentation of cross-border healthcare on both sides of the border, scientific analyses of the respective data and leadership of the political decision-makers to set the legal framework and administrative procedures. For instance, there is an urgent need to change the aviation laws of both countries so that ambulance helicopters can fly to emergencies on the other side of the border. Furthermore, the control centers of emergency units on both sides of the border still communicate (even in case of an emergency requiring forces from both sides of the border) via fax or telephone with each other because there is no unified radio communication system. Intensive research has to follow to improve the situation, but understanding the relevance of borders (as presented in this paper) is a good starting point.

Opening a political border will not necessarily change the situation. As shown in Sect. “Health impact”, opening the border might even lead to a situation that is worse than before opening the borders. Smaller hospitals might have to close down while some part of the population might be under-served with acute medical care. In the long run and in particular when the financial level of the countries get closer to each other, the situation will improve. However, at least for some decades the borders still exist mentally (see Sect. “Health impact”). This calls for a bundle of efforts to build up trust in the healthcare system of the neighbours, improve communication and overcome legal barriers. This could be based on several instruments. Firstly, quality management systems should be unified for hospitals on both sides of the border. Patients from the other side could, for instance, recognize a certificate (e.g., TÜV based on DIN EN ISO). This will increase their trust in the quality of services. However, quality management systems and research on quality management are frequently limited to one territory without considering trans-border acute care.

Secondly, hospital staff should be trained in the professional communicative competence of the other country's language [36]. For instance, the hospital in Wolgast closed down delivery services so that pregnant women from Eastern Usedom have to decide to travel to Greifswald, Anklam or to Swinoujscie. However, it is crucial that midwives in Swinoujscie hospital speak German if a German woman delivers there. This prerequisite is not fulfilled so that only very few women decide to seek services there. The question whether medical staff on both sides of the border should be able to communicate in English or the respective language of the other country (e.g., German and Polish), has to be addressed. Furthermore, it must be analyzed whether staff must be able to communicate in the foreign language or be conversant only in medical terms. There is a need for further research on patient preferences and training concepts of communication.

Thirdly, cross-border emergency care should be fostered. For this purpose, an EU-funded project was launched so that emergency paramedicals from both sides of the border train together and learn the language of the other country [37]. However, until today cross-border helicopter emergency flights are prohibited due to (seemingly) irreconcilable aviation laws. There is a strong need for an international treaty on cross-border emergency services. At the same time, there is a need for further research on cross-border emergency services. In particular, ambulances and helicopters are not the only emergency services in the border areas. Voluntary fire brigades, live guards on the beaches and first-responders of emergencies will all meet patients and victims from the other side of the border. To our knowledge, no structured research on the advantages, disadvantages, specific motivation, communication capabilities etc. of volunteers in cross-border emergency care exists.

The models presented in Sect. “Health impact” demonstrate that opening political and mental borders will not always results in an improved health situation for the population. It can happen that the catchment areas of some hospitals shrink so much that they cannot survive anymore. Thus, all attempts to increase cross-border healthcare (in particular emergency care) must be seen in the wider context of sustaining healthcare in rural areas with a focus on the special situation of small rural hospitals in rural peripheral areas [38]. It is an important step that rural healthcare is set on the political agenda in many nations. This will, eventually, be a chance to give more attention to cross-border acute care for the benefit of (potential) patients.
